# Chimeric VLPs Based on HIV-1 Gag and a Fusion Rabies Glycoprotein Induce Specific Antibodies against Rabies and Foot-and-Mouth Disease Virus

**DOI:** 10.3390/vaccines9030251

**Published:** 2021-03-12

**Authors:** Diego Fontana, Ernesto Garay, Laura Cervera, Ricardo Kratje, Claudio Prieto, Francesc Gòdia

**Affiliations:** 1 Cell Culture Laboratory, CBL (Biotechnological Center of Litoral), CONICET, FBCB (School of Biochemistry and Biological Sciences), UNL, S3000ZAA Santa Fe, Argentina; egaray@fbcb.unl.edu.ar (E.G.); rkratje@fbcb.unl.edu.ar (R.K.); 2 Biotechnological Development Laboratory, CBL (Biotechnological Center of Litoral), FBCB (School of Biochemistry and Biological Sciences), UNL, S3000ZAA Santa Fe, Argentina; cprieto@fbcb.unl.edu.ar; 3 Departament d’Enginyeria Química, Biològica i Ambiental, Universitat Autònoma de Barcelona, 08193 Bellaterra, Spain; Laura.Cervera@uab.cat (L.C.); Francesc.Godia@uab.cat (F.G.)

**Keywords:** virus-like particles, rabies, foot-and-mouth disease, HIV-1, chimeric, vaccine

## Abstract

Foot and mouth disease is a livestock acute disease, causing economic losses in affected areas. Currently, control of this disease is performed by mandatory vaccination campaigns using inactivated viral vaccines. In this work, we describe the development of a chimeric VLP-based vaccine candidate for foot-and-mouth disease virus (FMDV), based on the co-expression of the HIV-1 Gag protein and a novel fusion rabies glycoprotein (RVG), which carries in its N-term the FMDV main antigen: the G-H loop. It is demonstrated by confocal microscopy that both Gag-GFP polyprotein and the G-H loop colocalize at the cell membrane and, that the Gag polyprotein of the HIV virus acts as a scaffold for enveloped VLPs that during the budding process acquires the proteins that are being expressed in the cell membrane. The obtained VLPs were spherical particles of 130 ± 40 nm in diameter (analyzed by TEM, Cryo-TEM and NTA) carrying an envelope membrane that efficiently display the GH-RVG on its surface (analyzed by gold immunolabeling). Immunostainings with a FMDV hyperimmune serum showed that the heterologous antigenic site, genetically fused to RVG, is recognized by specific G-H loop antibodies. Additionally, the cVLPs produced expose the G-H loop to the liquid surrounding (analyzed by specific ELISA). Finally, we confirmed that these FMD cVLPs are able to induce a specific humoral immune response, based on antibodies directed to the G-H loop in experimental animals.

## 1. Introduction

Foot and Mouth Disease (FMD) is a livestock viral acute disease-causing important economic loss due to animal products trade restrictions and reduced productivity on affected countries. It is caused by a highly infectious and antigenically variable virus called Foot-and-Mouth Disease Virus (FMDV), member of the Picornaviridae family and Apthovirus genus. Its genome consists of a single-stranded RNA of positive polarity that encodes a polyprotein that is self-cleaved by a protease (3Cpro) giving rise to structural and non-structural proteins [1]. The capsid proteins VP1–4 assemble in capsomers forming a non-enveloped icosahedral particle. The most important antigenic site is an extremely flexible loop region named G-H loop, located on amino acids (AA) 140–160 of VP1 [2]. It is also the most variable region between different serotypes, with the exception of a highly conserved RGD motif known to interact with host cell integrins and responsible for virus entry into the cell [3]. To date, seven serotypes (A, O, C, Asia-1, SAT1, SAT2 and SAT3) and multiple subtypes have been reported (Diaz-San Segundo et al, 2017). 

Several measures are taken to control FMD in endemic areas, relying on high surveillance and massive vaccination of livestock, as well as strict control of animal movement in and out of monitored areas [4]. The implementation of massive vaccination was a key measure to eradicate the disease in affected countries of South America, and to control possible outbreaks. Currently used vaccines consist of inactivated FMDV formulated with various adjuvants, usually containing two or more serotypes or subtypes (Cao et al, 2016). Despite their efficacy to control the disease, this type of vaccines has several disadvantages: they require high biosafety environments for their production due to the fact that infective virus must be manipulated, they do not provide long term protection, require multiple doses, and must be maintained in cold chain as viral particles are thermo labile, having short shelf-life [5]. 

Lots of efforts are being directed to develop new generation vaccines that solve the difficulties previously mentioned, mostly toward subunit vaccines [5,6,7]. Virus-like particles (VLPs) are a very promising technology in that field, being supramacromolecular arrangements of viral proteins that mimic very closely the structure of the virus but lack the viral genome, making them biologically safe. They are highly immunogenic because of their particulated and highly multivalent nature, and can be obtained in a variety of expression systems as bacterial, yeast, plant, as well as insect and mammalian cells [8,9,10,11]. 

FMDV empty capsids are normally observed in vivo during infection and have been obtained by recombinant expression of FMDV polyprotein P1-2A and 3Cpro using the baculovirus-insect cell system and insect larvae, as well as in viral vectored vaccines and mammalian expression systems. These particles have proven to be effective at preventing animals from developing clinical symptoms upon FMDV exposure, with different degrees of protection [12]. Despite the protection induced in experimentation animals, recent studies on the immune response triggered by these particles have concluded that capsid integrity plays a key role in the development of a potent immune response in cattle [13], which is problematic for certain serotypes known to have less stable capsids [14]. On the other hand, recombinant empty capsid production is problematic because 3Cpro is toxic to the host cell and as these particles are not secreted, a cell disruption step must be added to the bioprocess [12]. These problems have been impairing the application of this technology to a larger production scale.

The Gag polyprotein from the HIV virus is able to self-assemble into particles without the need of any other viral protein. It accumulates at the vicinity of the producer cell membrane and buds taking part of the lipidic cell membrane and the proteins that are expressed at its surface [15]. The transient gene expression (TGE) of this protein, in mammalian cell lines using serum-free media, have been widely explored as an interesting platform for the production of biosafe vaccine candidates [16,17,18,19,20].

In this work, we designed a fusion protein based on the rabies virus (RV) glycoprotein and the FMDV G-H loop, to be co-expressed with the HIV-1 Gag capsid protein to express a chimeric VLP (cVLP) as a novel vaccine candidate for FMD. The Gag gene was fused in frame to the enhanced GFP gene, which allows for easy quantification of VLPs using fluorometry and nanoparticle tracking analysis (NTA) techniques and also facilitates characterization during process development. These functionalized Gag-based VLPs were completely characterized and the humoral immune response triggered in mice was studied. 

## 2. Materials and Methods

### 2.1. Fusion Protein Construction. Plasmids

The pGag-EGFP plasmid used, codes for a Rev independent HIV-1 Gag protein fused in frame to the enhanced GFP (Hermida-Matsumoto and Resh, 2000). The plasmid from the NIH AIDS Reagent Program (Cat 11468) was constructed by cloning the Gag sequence from pCMV55M1–10 (Schwartz et al, 1992) into the pEGFP-N1 plasmid (Clonthec, Mountain View, CA).

The coding sequence for the GH-RVG fusion protein was constructed inserting the G-H loop 63 bp sequence of FMDV A/Arg/2001 strain, between the RV signal peptide and the N-term extreme of the glycoprotein. This 1650 bp fragment was then cloned in pCiNeo expression vector (Promega, Madison, WI, USA), obtaining the pCiNeo- GH-RVG fusion protein plasmid.

### 2.2. Cell Line, Media, and Culture Conditions

The HEK293 cell line provided by Dr. Amine Kamen from McGill University (Montreal, Canada) derives from a cGMP master cell bank that is available for manufacturing of clinical material. It is adapted to grow in suspension and in serum free medium. Cells were cultured in Freestyle 293 medium (Invitrogen, Carlsbad, CA, USA) supplemented with 0.1% Pluronic® (Invitrogen, Carlsbad, CA, USA). Medium was also supplemented with 1.6 mg/L of r-transferrin (Merck Millipore, Kankakee, IL, USA), 19.8 mg/L of r-insulin (FeF Chemicals/Novo Nordisk, Køge, Denmark.), and 0.9X of an in-house lipid mixture [21]. Cells were routinely maintained in 125-mL disposable polycarbonate Erlenmeyer flasks (Corning, Steuben, NY, USA) in 20 mL of culture medium, shaken at 110 rpm using an orbital shaker (Stuart, Stone, UK) placed in an incubator maintained at 37 °C in a humidified atmosphere of 5% CO2 in air. Cell count and viability were determined using NucleoCounter NC-3000 (ChemoMetec, Allerod, Denmark).

### 2.3. Transient Transfection

HEK293 cells were seeded at 0.5 × 10^6^ cells/mL, allowed to grow for two days until they reached 2 × 10^6^ cells/mL and were transiently transfected using 25-kDa linear polyethyleneimine (PEI) (PolySciences, Warrington, PA). Transfections were performed using a final DNA concentration of 1 ug/mL of medium. PEI/DNA polyplexes were formed by adding PEI to plasmid DNA (in a ratio 2:1) diluted in fresh culture media. Complexes were incubated for 15 min at room temperature (RT) and added to the culture. When production enhancers were studied, valproic acid (3.36 mM) and caffeine (5.04 mM) were added to cell culture 4 hours post transfection (hpt). For analysis, transfected cells or culture medium containing VLPs were harvested at 24–96 hpt, depending on the experiment.

### 2.4. VLP Purification

24–96 hpt culture was harvested, centrifuged at 300× *g* for 10 min to remove cells and then at 10,000× *g* for 10 min to remove any remaining cellular debris. The clarified supernatant was layered over a 30% sucrose cushion and centrifuged at 100,000 g for 3 h at 4 °C (Beckman SW32 rotor, Beckman L100XP, Beckman Coulter, USA). The supernatant and sucrose cushion were poured off and the pellet was resuspended in PBS.

### 2.5. Confocal Microscopy

To analyze the expression of recombinant fusion proteins, 48-72 hpt freshly harvested cells were pelleted by centrifugation, resuspended in PBS and incubated for 30 min at RT with an in-house developed RVG specific monoclonal antibody [22] (diluted 1:1000 in PBS) or, alternatively, with a polyclonal antibody raised against FMDV, obtained immunizing rabbits with a commercial FMDV vaccine (Bioaftogen^®^, Biogénesis Bago, Argentina). Cells were washed (PBS), followed by 30 min incubation at RT with an AlexaFluor647^®^ conjugated anti-mouse or anti-rabbit antibody, respectively (Thermo Fischer Scientific, Waltham, MA, USA) diluted 1:000 in PBS). After that, cells were washed (PBS) and stained with Hoeschst and CellMask^TM^ (Thermo Fischer Scientific, Waltham, MA, USA) for 10 min at RT. After another wash, cells were resuspended in fresh FreeStyle 293 cell culture medium.

#### 2.5.1. Standard Confocal Laser Scanning Microscopy (CLSM)

Visualization of VLP producer cells was performed by Fluoview^®^ FV100 confocal microscope (Olympus, Tokyo, Japan). The excitation/emission parameters of each dye used for Confocal Microscopy were 633 nm/649 to 785 nm for Cell MaskTM, 405 nm/415 to 480 nm for Hoechst, 561 nm/580 to 630 nm for AlexaFluor647^®^ and 488 nm/500 to 560 nm for Gag-GFP VLPs. Samples were placed in glass bottom dishes (MatTek, Boston, MA) for visualization under the microscope. Final images were processed using LAS AFTM software (Leica Microsystems, Weztlar, Germany).

#### 2.5.2. Super-Resolution Fluorescence Microscopy (SRFM) 

SRFM analysis was performed using TCS SP8 confocal microscope equipped with Huygens deconvolution suite and GPU arrays (Leica Microsystems, Wetzlar, Germany) at Servei d’Anatomia Patològica from Hospital Sant Joan de Déu (Esplugues de Llobregat, Barcelona, Spain). VLPs were imaged with HC PL APO CS2 100x/1.40 OIL objective (zoom 5) with HyVolution2 (Lightning) mode (Leica Microsystems), 496 × 496–633 Hrz speed and line average of 3. 488 nm (Gag-eGFP VLPs) and 568 nm (mAB) laser lines and HyD detectors with BrightR mode (500–570 nm and 580–794 nm, respectively) were used. HyVolution2 deconvolution was performed with SVI Huygens Professional software using the best resolution strategy (Scientific Volume Imaging, Hilversum, The Netherlands).

### 2.6. Flow Cytometry

Transfected cells were fixed in 4% paraformaldehyde and then incubated for 30 min at RT with an in-house RVG protein specific monoclonal antibody (diluted 1:1000 in PBS) [22]. Cells were washed with PBS, followed by 30 min incubation at RT with an AlexaFluor647^®^ conjugated goat anti-mouse antibody (Thermo Fischer Scientific, Waltham, MA, USA) diluted 1:000 in PBS). Cells were washed and resuspended in PBS for flow cytometry analysis using a BD FACS Canto flow cytometer (BD Biosciences, San Jose, CA, USA).

### 2.7. VLPs Analysis and Quantitation

#### 2.7.1. Bi-specific” Sandwich ELISA 

96-well microplates (Greiner Bio-one, Kremsmünster, Austria) were coated with 100 μL of a 1:1000 dilution of a rabbit anti-FMDV serum in carbonate buffer pH 9.6 for 1 h at 37 °C and overnight at 4 °C. Plates were washed six times with PBS-Tween-20 0.05% and then blocked with 200 μL per well of 2% skim milk in PBS for 1 h at 37 °C. Two-fold serial dilutions of concentrated cVLP samples in skim milk 0.2%-PBS tween-20 0.05% were incubated for 1 h at 37 °C. After that, 100 μL of a 1:2000 diluted biotynilated rabbit anti-RVG serum in skim milk 0.2%-PBS tween-20 0.05% was added to each well and incubated for 1 h at 37 °C. Later, 100 μL of an HRP-streptavidin complex (AMDEX, Sigma-Aldrich, Saint Louis, MI, USA) diluted 1:10000 was added to each well and incubated for 1 h at 37 °C. Six washes with PBS-Tween-20 0.05% were done between each incubation. Finally, the reaction was revealed adding 100 μL of a chromogenic substrate solution to each well (0.5 mg.mL^−1^ o-phenylenediamine (Sigma-Aldrich), 0.5 μL.mL^−1^ H2O2 30 vol., 50 mM citrate/phosphate buffer, pH 5.3). The reaction was stopped adding 50 μL of a 0.5 M H2SO4 solution and the optical density was measured at 492 nm in a plate reader spectrophotometer (Labsystems Multiskan^®^).

#### 2.7.2. Fluorescence-Based Quantification of VLP Production

The concentration of cVLPs was assessed by fluorometry using an in-house developed and qualified quantification assay [23]. VLP containing supernatants were recovered by cell culture centrifugation at 300 g for 5 min. Green fluorescence was measured at RT using a Cary Eclipse Fluorescence Spectrophotometer (Agilent Technologies, Santa Clara, CA, USA) set as follows: λex = 488 nm (slit 5 nm), λem = 510 nm (slit 10 nm). Relative fluorescence unit values (RFU) were calculated by subtracting fluorescence units (FU) values of untransfected negative control samples. There is a linear correlation between fluorescence intensity and p24 values determined using the INNOTEST ELISA HIV antigen mAb (Innogenetics NV, Gent, Belgium). RFU values can be converted to Gag-GFP concentration values using the following equation:

Gag-GFP (ng/mL) = (3.245 × RFU) − 1.6833) × 36(1)

where Gag-GFP is the estimated concentration of polyprotein and RFU is the measured GFP fluorescence intensity in the samples. The first term is the correlation equation between fluorescence values and p24 concentrations determined by ELISA, and 36 is a correction factor that takes into account the difference in molecular weight between p24 and Gag-GFP and an underestimation arising from using the p24 ELISA to estimate p55 Gag concentrations.

#### 2.7.3. Nanoparticle Tracking Analysis (NTA)

A NanoSight^®^ NS300 device (Malvern Panalytical, Malvern, United Kingdom) equipped with a blue laser module (488 nm) for fluorescent particles (Gag-GFP VLPs), and a neutral density filter for total nanoparticle measurement using light diffraction was used. The measurements were performed at Service of Preparation and Characterization of Soft Materials (Institut de Ciència de Materials de Barcelona, ICMAB-CSIC, Cerdanyola del Vallès, Spain). Data were analyzed with the NanoSight^®^ NTA 3.2 software (Malvern Panalytical) and the camera level and detection threshold were manually adjusted for each sample. Before injection in the device chamber, samples were diluted to a final volume of 1 mL with a nanoparticle concentration around 10^8^ particles/mL. All measurements were performed in triplicate and normalized by an internal control consisting of HIV-1 Gag-eGFP VLPs.

### 2.8. Electron Microscopy

#### 2.8.1. Transmission Electron Microscopy (TEM)

cVLPs were adsorbed onto formvar-coated 300-mesh copper grids for 2 min. Excess was removed with filter paper and grids were then negatively stained with 2% uranyl acetate for 2 min. Samples were observed in a Jeol JEM-1400 (Jeol LTD, Tokyo, Japan) TEM equipped with a CCD-multiscan camera (Model No. 895, Gatan Inc., Pleasanton, CA, USA).

For TEM immunolabeling assays, after VLPs adsorption grids were blocked with 2% BSA in PBS, they were then incubated with an in-house developed RVG protein specific monoclonal antibody [22] (diluted 1:100 in PBS). Cells were washed (PBS), followed by 30 min incubation at RT with with secondary goat anti-mouse antibody (at a dilution of 1 in 25) coupled with 15 nm gold particles (15 nm, BBI Solutions, Cardiff, Wales, UK).

#### 2.8.2. Cryogenic Electron Microscopy (CryoTEM)

cVLP morphology was studied by CryoTEM. A 2–3 μL amount of sample was blotted onto holey carbon grids (Quantifoil Micro Tools, Großloebichau, Germany and Micro to Nano, Haarlem, Netherlands) previously glow discharged in a PELCO easiGlow glow discharger unit. The samples were subsequently plunged into liquid ethane at −180 °C using a Leica EM GP cryo workstation and observed in a JEM 2011 electron microscope (JEOL Ltd., Tokyo, Japan) operating at 200 kV. During imaging, samples were maintained at −173 °C, and pictures were taken using a CCD-multiscan camera (Model No. 895, Gatan Inc., Pleasanton, CA, USA).

### 2.9. Mice Immunization and Serology

#### 2.9.1. Immunization Plan

The immunization protocols were approved and supervised by the Advisory Committee on Ethics and Security of the School of Biochemistry and Biological Sciences, Universidad Nacional del Litoral, according to international guidelines (“Guide for the Care and Use of Laboratory Animals”, Eighth Edition National Research Council 2011).

Female Balb/c mice of 6–8 weeks of age (SPF, Centro de Medicina Comparada, ICIVET-CONICET UNL, Argentina) were intramuscularly immunized with three doses at days 0, 14 and 35. In group I (*n* = 7) animals were immunized with GH-RVG/Gag-GFP chimeric VLPs (cVLPs), purified by ultracentrifugation. Each dose contained a total amount of 2.8 × 10^10^ particles in 100 uL of PBS, measured by fluorometry. This corresponds to 0.24 Elisa Units per dose of rabies glycoprotein, measured by sandwich ELISA [24]. In group II (*n* = 8) mice were injected with VLPs composed only of RVG (RVG VLPs), produced as was previously described [24,25]. For this group, each dose contained 0.24 Elisa Units of rabies glycoprotein in 100 ul of PBS, i.e, a dose equivalent to that the given in group I. Both VLPs were formulated with LipoSap^®^ adjuvant with a final concentration of 100 μg/mL. This adjuvant was commercially obtained from Lipomize S.R.L., Argentina (www.lipomize.com.ar) and is the commercial product of an adjuvant called ISPA [26,27], which consists of cage-like particles with low surface charge density containing Quil—A^®^ as immune response stimulator. This adjuvant was previously evaluated for FMDV and rabies VLPs, as vaccine candidates [27,28,29]. As a control or reference, a commercial anti-FMDV vaccine (Bioaftogen^®^, Biogénesis Bagó, Argentina) was injected in a third group (*n* = 8), with 20 μL dose per animal. Animals were bled two weeks after the last dose and the sera was obtained and conserved at −20 °C until further analysis.

#### 2.9.2. Quantification of RV Specific Immunoglobulins

96-well microplates (Greiner Bio-one, Kremsmünster, Austria) were coated with 100 μL of an appropriate dilution of RVG-VLPs in carbonate buffer pH 9.6 for 1 h at 37 °C and overnight at 4 °C. Plates were washed six times with PBS-Tween-20 0.05% and then blocked with 200 μL per well of 2% skim milk in PBS for 1 h at 37 °C. Two-fold serial dilutions of sera samples in skim milk 0.2%-PBS Tween-20 0.05% were incubated for 1 h at 37 °C. After that, 100 μL of an HRP conjugated anti-mouse antibody (Dako, Agilent Technologies, Santa Clara, CA, USA) diluted 1:1000 in skim milk 0.2%-PBS tween-20 0.05% was added to each well and incubated for 1 h at 37 °C. A total of six washes with PBS-Tween--20 0.05% were done between each incubation. Finally, the reaction was revealed adding 100 μL of a chromogenic substrate solution to each well (0.5 mg.mL^−1^ o-phenylenediamine (Sigma-Aldrich, Saint Louis, MI, USA), 0.5 μL/mL H_2_O_2_ 30 vol., 50 mM citrate/phosphate buffer, pH 5.3). The reaction was stopped adding 50 μL of a 0.5 M H_2_SO_4_ solution and the optical density was measured at 492 nm in a plate reader spectrophotometer (Labsystems Multiskan^®^). Antibody titers were calculated as the end-point serum dilution yielding an optical density higher than the cut-off value. This cut-off was calculated as the mean + 2 S.D. of the optical density of negative controls (basal mice sera).

#### 2.9.3. Quantification of FMD Disease Epitope Specific Immunoglobulins

96-well microplates (Polysorp^®^, NuncTM) were coated with 2 μg per well of a G-H loop A/Arg/01 synthetic peptide (GSSRRGDLGSLAARVVKALPA, GenScript^®^, NJ, USA) in carbonate buffer pH 9.6 for 1 h at 37 °C and overnight at 4 °C. Plates were washed six times with PBS-Tween-20 0.05% and then blocked with 200 μL per well of 2% skim milk in PBS for 1 h at 37 °C. Two-fold serial dilutions of sera samples in skim milk 0.2%-PBS tween-20 0.05% were incubated for 1 h at 37 °C. After that, 100 μL of an anti-mouse rabbit serum diluted 1:500 in skim milk 0.2%-PBS tween-20 0.05% was added to each well and incubated for 1 h at 37 °C. After that, an HRP conjugated anti-rabbit antibody (Dako, Agilent Technologies, Santa Clara, CA, USA) diluted 1:1000 in skim milk 0.2%-PBS tween-20 0.05% was added to each well and incubated for 1 h at 37 °C. A total of six washes with PBS-Tween-20 0.05% were done between each incubation. Finally, the reaction was revealed adding 100 μL of a chromogenic substrate solution to each well (0.5 mg.mL^−1^ o-phenylenediamine (Sigma-Aldrich, Saint Louis, MI, USA), 0.5 μL.mL^−1^ H2O2 30 vol., 50 mM citrate/phosphate buffer, pH 5.3). The reaction was stopped, adding 50 μL of a 0.5 M H2SO4 solution and the optical density was measured at 492 nm in a plate reader spectrophotometer (Labsystems Multiskan^®^). Antibody titers were calculated as the end-point serum dilution yielding an optical density higher than the cut-off value. This cut-off was calculated as the mean + 2 S.D. of the optical density of negative controls (basal mice sera).

### 2.10. Statistical Analysis

Differences between treatments were evaluated through a one-way analysis of variance (ANOVA). When the ANOVA produced significant results (*p* < 0.05), a post-hoc Tukey’s multiple comparison test was applied. All statistical analyses were performed using GraphPad Prism for Windows, version 5.01 (GraphPad Software Inc.).

## 3. Results

### 3.1. Design, Construction and Analysis of the Expression of GH-RVG Fusion Protein

**Figure 1:**
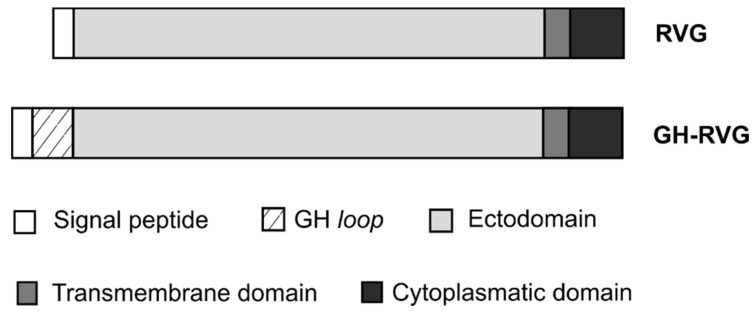
GH-RVG fusion protein structure. The coding sequence of the FMDV GH loop epitope was inserted between the glycoprotein signal peptide and the ectodomain. RVG: native rabies virus glycoprotein.

With the objective to construct a rabies fusion glycoprotein displaying the G-H loop epitope on the surface of the cVLPs, the sequence of the corresponding 21 amino acids was inserted in the N-term of RVG, after the signal peptide (Figure 1). This sequence was cloned in pCiNeo plasmid, obtaining the pCiNeo-GH-RVG expression vector.

**Figure 2:**
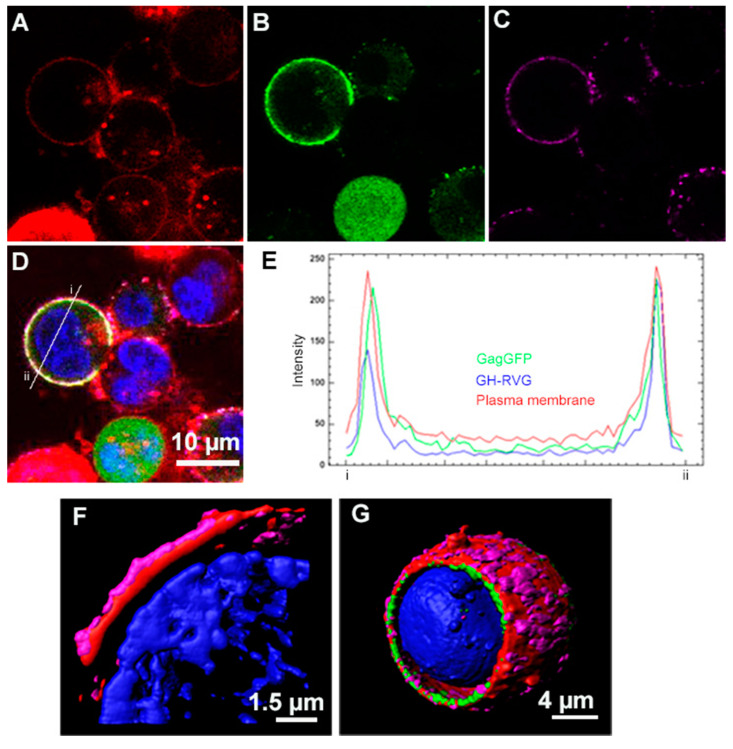
GH-RVG fusion protein expression analysis. HEK293 cells were co-transfected with GH-RVG and Gag-GFP vectors, and 72 hpt were incubated with an in-house developed RVG specific monoclonal antibody, followed by an incubation with an AlexaFluor647^®^ conjugated anti-mouse secondary antibody (magenta). Lipid membrane was stained with CellMask™ (red) and cell nucleus was stained with Hoechst (blue). The green signal corresponds to Gag-eGFP (B) and D is the merge of the four channels. (**A**–**D**): Standard confocal laser scanning microscopy. (**E**): Fluorescence intensity though the diameter of the cell is represented with a histogram performed with FIJI (Image J) software. Green line corresponds to GagGFP, red line to the CellMask staining and the blue line correspond to the fluorescence intensity obtained with the immunolabeling of the cells (AlexaFluor647). (**F**,**G**): Super-resolution fluorescence microscopy. (**F**): expression of GH-RVG fusion glycoprotein alone. (**G**): Co-expression of GH-RVG and Gag-GFP fusion proteins.

In order to analyze if the insertion of this epitope had modified the correct expression and the subcellular localization of the RVG protein, using pCiNeo-GH-RVG and pGag-EGFP plasmids, we performed a co-transfection of HEK293 cells. At 72 hpt, we analyzed the cells by standard confocal laser scanning microscopy (CLSM). As can be seen in Figure 2, the fluorescence corresponding to the anti-rabies antibody immunostaining was detected on the cell surface (Figure 2C), showing that GH-RVG is anchored in the plasma membrane, as expected for the rabies glycoprotein [30]. On the other hand, when GH-RVG was co-expressed with Gag-GFP we observed a clear overlapping of the signals, matching cell membrane staining signal (Figure 2D,E). These results showed that both chimeric proteins are found in the plasma membrane of the cell, from where VLPs bud to the supernatant [31].

Later, these observations were confirmed by super-resolution fluorescence microscopy (SRFM). With this novel technique higher resolutions are obtained when viral chimeric protein are analyzed, both within the cell during VLP budding or at the single particle analysis [15]. We could clearly observe how GH-RVG fusion protein is exposed in the outer part of the transfected cell while Gag-GFP is associated with the inner part of the plasma membrane (Figure 2F,G). In addition, GH-RVG protein is concentrated in clusters throughout the cell surface, presumably associated with lipid rafts, as described for the rabies glycoprotein and other viral membrane proteins [32,33]. These results demonstrated that the insertion of the GH-loop epitope within the RVG sequence did not alter the normal pathway for the expression of this glycoprotein or its folding.

**Figure 3:**
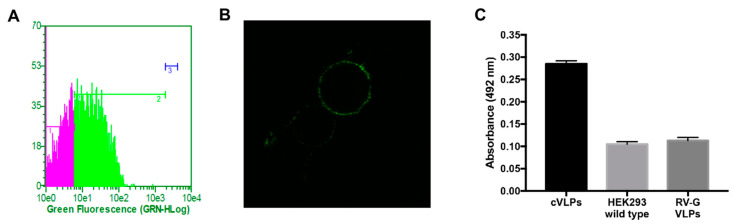
G–H loop epitope detection. A–B: HEK293 cells were transfected with GH-RVG vector and, 72 hpt, cells were incubated with an in-house developed FMDV specific polyclonal antibody, followed by an incubation with an AlexaFluor488^®^ conjugated anti-rabbit secondary antibody. (**A**): Flow cytometry. Marker 1 represents negative cells and marker 2 and 3 the positive cells. (**B**): Confocal microscopy. (**C**): HEK293 were co-transfected with GH-RVG and Gag-GFP vectors, and 72 hpt cVLPs were harvested and purified by ultracentrifugation. The exposition of the GH loop epitope in this VLPs sample was analyzed by bi-specific ELISA: cVLPs were captured using a FMDV specific serum and the detection was performed using an anti-RV antibody. As controls, we used culture medium of wild type HEK293 cells and VLPs containing wild type RVG (without the FMDV epitope). Results are expressed as mean ± SD (*n* = 3).

Next, we investigated if in the context of the GH-RVG fusion protein, the foot-and-mouth disease virus (FMDV) epitope was correctly exposed to the liquid surrounding, and able to be recognized by specific antibodies. To study this, cells transfected only with GH-RVG vector were immunostained with a polyclonal antibody specific for FMDV, and analyzed by flow cytometry and SCLM (Figure 3A,B). As can be seen in Figure 3A, 72 hpt, the heterologous epitope present in GH-RVG fusion protein, was able to be detected by specific antibodies (marker 2). In addition, when cells were observed in SCLM (Figure 3B), the specific fluorescence was detected on the cell surface, as was previously observed when cells were analyzed with anti-rabies antibodies. This result is of paramount importance since we hypothesized that, if this chimeric protein is able to correctly expose the GH-loop epitope, being able to interact with anti-FMDV antibodies in solution, it might also be able to induce the production of anti-epitope antibodies when injected as an immunogen. This interpretation was later confirmed in this work.

### 3.2. cVLP Expression and Characterization

**Figure 4:**
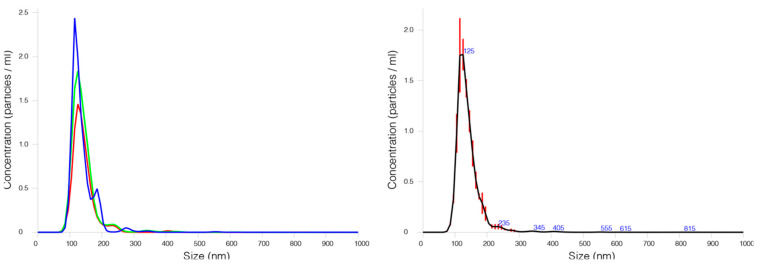
Particle size distribution cVLPs. 72 hpt medium culture was harvest and clarified by 0.45 µm filtration. Fluorescent cVLPs were analyzed by nanoparticle tracking analysis using a NanoSight^®^ NS300 device equipped with a blue laser module (488 nm). In the left part of the figure, three independent measures are graphed and, in the right, the average particle size distribution of the fluorescent cVLPs present in the supernatant is showed.

Once the correct expression and subcellular localization of the GH-RVG fusion protein was confirmed, we analyzed the budding of cVLPs when HEK293 cells are co-transfected with both chimeric viral proteins, the HIV-1 Gag protein fused to the GFP and the GH-RVG. At 72 hpt, culture medium was harvested and the hydrodynamic diameter and particle size distribution of cVLPs were studied by nanoparticle tracking analysis (NTA) (Figure 4). 

Generally, NTA is a technique that detects and measures all particles present in the sample, where host cell debris and other extracellular particles can affect the correct size measurement of VLPs [34]. To overcome this problem, in this work, cVLPs were analyzed using a blue laser module, where only fluorescent particles (GFP positive) were quantified, obtaining an average value of 130 ± 40 nm with a particle size distribution similar to that previously described for Gag-based VLPs (Figure 4) [15].

**Figure 5:**
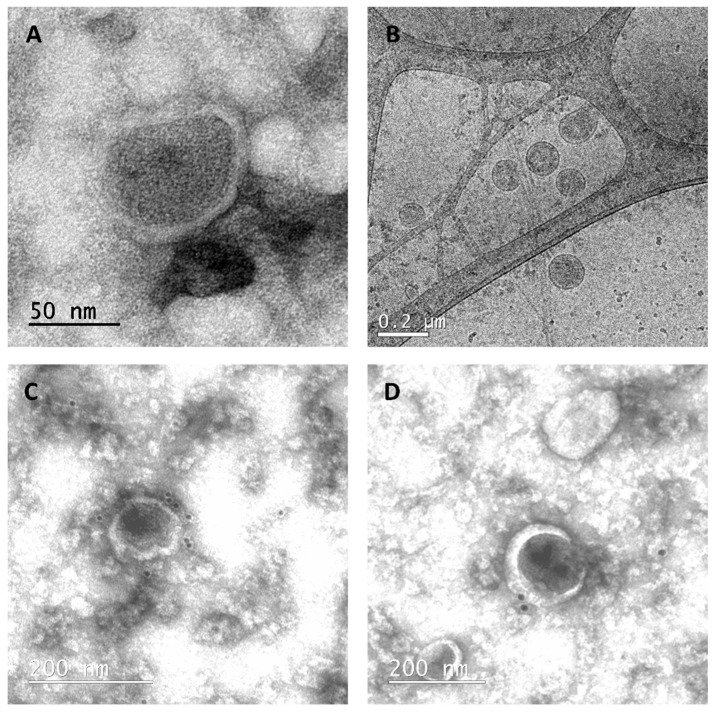
Electron microscopy analysis of cVLPs. 72 hpt cVLPs were harvested and purified by ultracentrifugation, before morphology analysis. (**A**): TEM of purified cVLPs. (**B**): CryoTEM. (**C,D**): Gold-immunolabeled TEM. Grids were incubated with a RVG protein specific monoclonal antibody, followed by an incubation a with secondary goat anti-mouse antibody coupled with 15 nm gold particles.

Further, cVLPs were purified by ultracentrifugation and their morphology was analyzed by transmission electron microscopy (TEM) and cryogenic electron microscopy (Cryo-TEM). As can be seen in Figure 5A,B, we observed enveloped spherical electrodense nanoparticles, as was previously described for GFP-Gag-based VLPs [15,35,36].

On the other hand, we investigated if the fusion protein GH-RVG was present in the structure of the observed particles, anchored in the VLPs envelope as is described when a membrane glycoprotein is co-expressed with Gag protein [37,38,39,40]. Therfore, we performed immunogold labelling of the purified cVLPs and analyzed it by TEM (Figure 5C,D). As can be observed in two individual fields, gold nanoparticles were detected surrounding the cVLP, specifically linked to the particle lipid membrane, revealing that the rabies chimeric fusion glycoprotein is exposed on the pseudoviral particles surface, as expected. In addition, in order to confirm if the G-H loop epitope was exposed on the VLPs and is able to be recognized by antibodies in solution, a preparation of purified cVLPs was analyzed by a bi-functional ELISA assay (Figure 3C). In this assay, VLPs were captured using a FMDV specific serum and the detection was performed using an anti-RV antibody. As can be seen in Figure 3C, a specific signal was detected in the cVLPs sample when compared with the controls, which means that in the context of this chimeric VLP, the G-H loop epitope is exposed and available to be recognized and bound by specific anti-FMDV antibodies.

### 3.3. cVLPs Production Optimization

The addition of valproic acid and caffeine increases cell viability and VLPs production of HIV-1 Gag based VLPs in HEK293 suspension cell cultures by 3.8-fold, as demonstrated previously [41]. In this work, we evaluated if these additives have the same positive effect in the expression of the rabies fusion protein and in the production of Gag VLPs functionalized with this envelope glycoprotein. 

**Figure 6:**
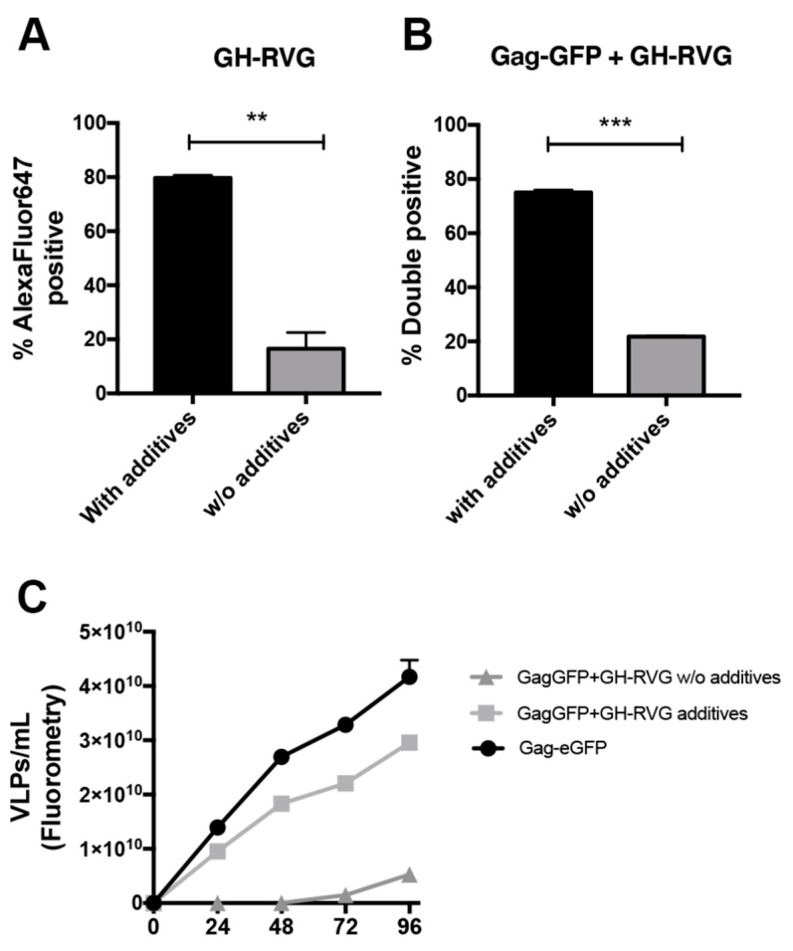
cVLPs production optimization. HEK293 cells were transfected with GH-RVG (**A**) or co-transfected with GH-RVG and Gag-GFP vectors (**B**), and 4 hpt valproic acid and caffeine (additives) were added to the culture, when correspond. 72 hpt cells were harvested and incubated with an in-house developed RVG specific monoclonal antibody, followed by an incubation with an AlexaFluor647^®^ conjugated anti-mouse secondary antibody. Finally, immunostained cells were analyzed by flow cytometry. (**C**): Medium culture of transfected cells was harvested each 24 h and cVLPs concentration was quantified by fluorometry. A single transfection with Gag-GFP alone was performed as a control, with additives. Results are expressed as mean ± SD (*n* = 3). ** *p* < 0.01. *** *p* < 0.001.

With this aim, we performed single (GH-RVG only) and double transfections (GH-RVG + Gag-GFP) of HEK239 cells and 4 hpt valproic acid and caffeine were added to the culture medium. On the one hand, 72 hpt cells were analyzed by flow cytometry in order to measure the percentage of GH-RVG positive cells (Figure 6A) or double positive cells (Figure 6B), both with or without additives. We observed that the expression level was increased by four-fold when additives were present in the culture medium after transfection.

These results show that caffeine and valproic acid have the same positive effect on the expression of the fusion glycoprotein than on the expression of Gag-GFP, as it has been previously reported [17,41]. On the other hand, we studied the effect of these additives on the concentration of cVLPs secreted (Figure 6B). As can be seen, additives have a clear positive effect on cVLPs production and budding, from the first 24 hpt through all the time period studied. Further, at 96 hpt, a culture without additives presented a particle concentration of 5.2 × 10^9^ VLPs/mL while when valproic acid and caffeine were added, we obtained a concentration of 2.9 × 10^10^ VLPs/mL, a 5.5-fold increase.

### 3.4. Evaluation of cVLPs Immunogenicity in Mice

**Figure 7:**
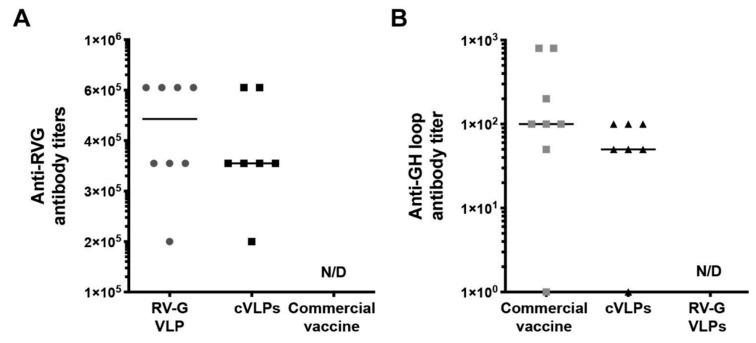
Humoral immune response triggered by cVLPs. Mice were vaccinated with three doses of cVLPs with adjuvant (*n* = 7). Two control groups were injected with rabies VLPs (RV-G VLPs) or a commercial FMDV vaccine (*n* = 8), following the same immunization protocol. Sera was obtained 15 days after the third dose and analyzed for (**A**): anti rabies specific antibodies, by indirect ELISA. (**B**): anti GH-loop FMDV epitope specific antibodies, by indirect ELISA using a synthetic peptide to coat the plates. Antibody titers were calculated as the end-point serum dilution yielding an optical density higher than the cut-off value. This cut-off was calculated as the mean + 2 S.D. of the optical density of negative controls (basal mice sera). Results are expressed as the individual titer values for each animal (dots) and the median of each group. N/D: not detectable.

Finally, after characterization of the constructed fusion glycoprotein and chimeric VLPs, confirming that the heterologous FMDV epitope was correctly expressed and exposed on the particle surface, we investigated if these cVLPs were able to induce a specific immune response against the G-H loop. Thus, we vaccinated mice with three successive doses of purified VLPs and, 15 days after the third dose, sera were obtained to analyze the humoral immune response triggered. First, anti-rabies immunoglobulins titer was quantified by indirect ELISA (Figure 7A), observing that mice injected with the cVLPs presented a titer comparable to that obtained in the group vaccinated with rabies VLPs (RV-G VLPs). This result showed that, although the primary structure of RVG was genetically modified by adding the G-H loop epitope, the GH-RVG fusion protein present in the cVLPs is still able to induce specific anti-rabies antibodies. Secondly, and more importantly, we analyzed the induction of antibodies against the heterologous FMDV epitope, performing an indirect ELISA developed to this aim. As can be seen in Figure 7B, when mice were injected with the cVLPs, specific anti-G-H loop antibodies were detected and quantified, with no statistical differences with the titer triggered by a commercial FMDV vaccine. In addition, no antibodies were detected in the group of mice injected with RV-G VLPs, demonstrating that this ELISA assay was efficient to detect only anti-GH loop antibodies.

These results demonstrated that the FMDV epitope present in the GH-RVG fusion protein is able to interact with the B cells receptors of the vaccinated animals, and induce the production of specific antibodies. Nevertheless, more experiments should be performed in order to study in more detail the immune response triggered.

## 4. Discussion

Foot-and-mouth disease is a viral relevant disease, both for animal health and for its economic impact, with the potential of producing millions of losses in livestock production. Currently, the main strategy to control the disease in endemic countries are mandatory vaccination campaigns, using inactivated viral vaccines [4,6,42,43]. Although these vaccines have been shown to be effective, the increment of worldwide trade, together with fast population growth, international travel and migration may contribute to the disease resurgence, even in countries where the disease is controlled without vaccination. This scenario will challenge the capabilities of the available vaccines triggering the need for improved vaccines against FMDV. New generation vaccine candidates, based on recombinant technologies, should play a central role in the search for improved FMDV vaccines, with higher potency, safety and DIVA (differentiating infected from vaccinated animals) characteristics [44].

Virus-like particles have been widely used for vaccine development in the last decades. Several VLP-based vaccines are commercially available for human use, including Human Papilloma Virus, Hepatitis B virus and, more recently, the first VLP-based malaria vaccine Mosquirix^®^ has been approved in Europe [8,11,45,46,47,48]. On the other hand, VLPs are being taken into account as potential immunogens for veterinary vaccines [49,50,51,52].

In this work, we described the development and analysis of a chimeric VLP-based vaccine candidate for FMDV, based on the co-expression, in HEK293 cells, of the HIV-1 Gag protein and a novel fusion rabies glycoprotein, that carries in its N-term the FMDV main antigen: the G-H loop (Figure 1). The main goal was to take advantage of the well stablished Gag polyprotein platform [16], together with optimized transient transfection techniques [17,41], in order to produce VLPs functionalized with the chimeric fusion protein (GH-RVG).

As a first step, we evaluated the cellular expression of GH-RVG by flow cytometry and confocal microscopy, demonstrating that the genetic fusion of G-H loop did not alter the correct sub-cellular localization of the native RVG (Figure 2). In addition, using new generation super-resolution imaging techniques, we showed that GH-RVG is localized in the plasma membrane of the transfected cells, concentrated in clusters, with the normal pattern described for this Rhabdovirus glycoprotein [32,33]. Further, when GH-RVG is co-expressed with Gag-GFP, co-localization of both proteins in the cell external membrane was observed (Figure 2). This result is crucial for the understanding of the cVLP budding process, as was recently described for naked Gag-GFP particles formation [35]. In this case, the merge of the three staining markers (magenta for GH-RVG, red for plasma membrane and green for Gag-GFP; Figure 2) shed us light on these functionalized VLPs morphogenesis, demonstrating that the pseudovirus particle assembly occurs in the cell plasma membrane. More importantly, performing immunostainings with a FMDV hyperimmune serum, we showed that the heterologous antigenic site, genetically fused to RVG, is available to be recognized by specific G-H loop antibodies (Figure 3A,B).

Later, we analyzed the cVLPs production when HEK293 are transfected with both viral proteins sequences, GH-RVG and Gag-GFP. First, by NTA, we analyzed the particle size distribution of cVLPs in the culture medium and calculated a hydrodynamic diameter of 130 ± 40 nm (Figure 4). After that, cVLPs were purified by ultracentrifugation and studied by TEM and Cryo-TEM. Spherical pseudo-viral particles were detected, carrying an envelope membrane that efficiently display the fusion glycoprotein on its surface (Figure 5). Additionally, we confirmed that the cVLPs are able to expose the G-H loop to the liquid surrounding when analyzed by specific ELISA (Figure 3B). 

Further, to obtain the proof-of-concept of these cVLPs as a FMDV vaccine candidate, we evaluated the immunogenicity of these chimeric particles in experimental animals. After immunizing mice with three successive doses at days 0, 14 and 35 (2.8 × 10^10^ VLPs per dose), we confirmed that these cVLPs are able to induce a specific humoral immune response based on antibodies directed to the G-H loop (Figure 7B), a key result showing that this FMDV domain is available to be recognized by the B cells receptors. Anti G-H loop antibodies have been proven to be the main responsible of FMDV neutralization and, thus, generate protection against virus infection [2,43,53,54]. Although these are preliminary results, they are very promising and encourage further studies to deeply characterize the immune response induced by these novel cVLPs, analyze the cellular response triggered as well and evaluate if it is able to protect animals from a virus challenge.

An interesting feature of this cVLPs is that they are able to trigger anti-rabies antibodies production (Figure 7A), together with the already discussed G-H loop specific antibodies. This is important considering that rabies and FMD are important zoonotic diseases that affect cattle, and the vaccination against these viruses are both mandatory in many countries, with annual re-vaccination schedules [5,55]. The possibility to have a vaccine that protect animals for both infections could be a good approach in order to reduce the overall vaccine price and simplify the vaccination logistics.

On the other hand, we worked on the bioprocess optimization in order to increase the cVLPs productivity. Applying a previously developed methodology for the improvement of Gag-GFP VLPs [19,41], we obtained a 5.5-fold increase in the VLPs yield (Figure 6). These results are of paramount importance considering that one of the main goals in the development of a vaccine candidate is the reduction for producing a vaccine dose. This becomes even more important when the vaccine is for veterinary applications, where prices are low and the achievements in the optimization of the process would have a higher impact. Furthermore, this TGE technique can be applied not only in batch and fed-batch processes, but to continuous VLPs production in bioreactor with perfusion, offering a platform for the future process development and scale up of this vaccine candidate production [56,57]. In any event, it is important to be aware that TGE present several challenges for its scaling-up process, including the large-scale high-quality DNA production and the need to prepare the DNA/polyplexes before their addition into the bioreactor, where large volume manipulation can cause delays in complex formation, impacting on the complex size distribution and, consequently, on the transfection efficiency. In addition, some TGE procedures include a medium exchange step, whereby scaling-up this is labor-intensive and associated with potential contamination risk. Nevertheless, in the last few years, great advances have been achieved in this field [20], which allow us to propose this culture platform for the production of a novel FMDV vaccine candidate. 

In conclusion, we consider that the results presented in this work represent a valid approach to the development of a novel recombinant FMDV vaccine candidate, avoiding virus manipulation during production, with no requirement for viral contention facilities. Furthermore, the results also show the capacity of Gag-VLPs as a platform to incorporate antigens from unrelated diseases. This enables the use of the already developed and optimized bioprocess for the Gag-VLP production to give a rapid response to emerging diseases or pandemic outbreaks only co-transfecting with the desired epitope. Additionally, in this work, we have demonstrated the easy production of the Gag-based VLPs pseudotyped with the GH-RVG fusion protein carrying the A/Arg/01 G-H loop serotype. As the FMDV has seven serotypes and the approved vaccines normally comprises two, three or four serotypes (depending on the country), this platform could be used to produce it in an easier manner (by co-transfecting with the plasmids encoding for the epitopes of the desired serotypes) than the actual production system based on the inactivated virus in which the different serotypes need to be produced separately, inactivated and mixed for the final vaccine. 

Concluding, these results encourage further studies to characterize the immune response triggered by these cVLPs and to work in the scaling-up process to evaluate the productivities that can be obtained.

## Data Availability

The data generated in this study are contained in this paper.
